# Galectin-1-mediated MET/AXL signaling enhances sorafenib resistance in hepatocellular carcinoma by escaping ferroptosis

**DOI:** 10.18632/aging.204867

**Published:** 2023-07-11

**Authors:** Tung-Wei Hsu, Yen-Hao Su, Hsin-An Chen, Po-Hsiang Liao, Shih Chiang Shen, Kuei-Yen Tsai, Tzu-Hsuan Wang, Alvin Chen, Chih-Yang Huang, Marthandam Asokan Shibu, Wan-Yu Wang, Shing-Chuan Shen

**Affiliations:** 1 Graduate Institute of Medical Sciences, College of Medicine, Taipei Medical University, Taipei 11031, Taiwan; 2 Division of General Surgery, Department of Surgery, Shuang Ho Hospital, Taipei Medical University, New Taipei City 23561, Taiwan; 3 TMU Research Center of Cancer Translational Medicine, Taipei Medical University, Taipei 11031, Taiwan; 4 Division of General Surgery, Department of Surgery, School of Medicine, College of Medicine, Taipei Medical University, Taipei 11031, Taiwan; 5 TMU Research Center for Digestive Medicine, Taipei Medical University, Taipei 11031, Taiwan; 6 Graduate Institute of Clinical Medicine, College of Medicine, Taipei Medical University, Taipei 11031, Taiwan; 7 Metabolic and Weight Management Center, Shuang Ho Hospital, Taipei Medical University, New Taipei City 23561, Taiwan; 8 Cardiovascular and Mitochondrial Related Disease Research Center, Hualien Tzu Chi Hospital, Buddhist Tzu Chi Medical Foundation, Hualien 97002, Taiwan; 9 Center of General Education, Buddhist Tzu Chi Medical Foundation, Tzu Chi University of Science and Technology, Hualien 97002, Taiwan; 10 Department of Medical Research, China Medical University Hospital, China Medical University, Taichung 404, Taiwan; 11 Graduate Institute of Biomedical Sciences, China Medical University, Taichung 404, Taiwan; 12 Department of Biotechnology, Bharathiar University, Coimbatore 641046, Tamil Nadu, India; 13 Department of Dermatology, School of Medicine, College of Medicine, Taipei Medical University, Taipei 11031, Taiwan; 14 International Master/PhD Program in Medicine, College of Medicine, Taipei Medical University, Taipei 11031, Taiwan

**Keywords:** Galectin-1, sorafenib resistance, ferroptosis, hepatocellular carcinoma

## Abstract

Sorafenib, a small-molecule inhibitor targeting several tyrosine kinase pathways, is the standard treatment for advanced hepatocellular carcinoma (HCC). However, not all patients with HCC respond well to sorafenib, and 30% of patients develop resistance to sorafenib after short-term treatment. Galectin-1 modulates cell-cell and cell-matrix interactions and plays a crucial role in HCC progression. However, whether Galectin-1 regulates receptor tyrosine kinases by sensitizing HCC to sorafenib remains unclear. Herein, we established a sorafenib-resistant HCC cell line (Huh-7/SR) and determined that Galectin-1 expression was significantly higher in Huh-7/SR cells than in parent cells. Galectin-1 knockdown reduced sorafenib resistance in Huh-7/SR cells, whereas Galectin-1 overexpression in Huh-7 cells increased sorafenib resistance. Galectin-1 regulated ferroptosis by inhibiting excessive lipid peroxidation, protecting sorafenib-resistant HCC cells from sorafenib-mediated ferroptosis. Galectin-1 expression was positively correlated with poor prognostic outcomes for HCC patients. Galectin-1 overexpression promoted the phosphorylation of AXL receptor tyrosine kinase (AXL) and MET proto-oncogene, receptor tyrosine kinase (MET) signaling, which increased sorafenib resistance. MET and AXL were highly expressed in patients with HCC, and AXL expression was positively correlated with Galectin-1 expression. These findings indicate that Galectin-1 regulates sorafenib resistance in HCC cells through AXL and MET signaling. Consequently, Galectin-1 is a promising therapeutic target for reducing sorafenib resistance and sorafenib-mediated ferroptosis in patients with HCC.

## INTRODUCTION

Liver cancer is the most common malignant neoplasm worldwide [1]. Approximately 90% of patients with liver cancer have hepatocellular carcinoma (HCC) [2]. Liver resection and transplantation are the main treatments for early-stage HCC. However, HCC is often detected at an advanced stage. For advanced-stage HCC, treatment with sorafenib is recommended [3, 4]. Sorafenib, an orally active multikinase inhibitor, is a potent first-line drug approved by the Food and Drug Administration for the treatment of advanced HCC [5]. However, approximately 30% of patients develop resistance to sorafenib [6]. Therefore, molecular mechanisms underlying sorafenib resistance in patients with HCC should be urgently determined.

Galectins are a family of β-galactoside-binding proteins. Among them, Galectin-1 is involved in the regulation of cell–cell and cell–matrix interactions and multiple aspects of cancer progression and tumor biology [7, 8]. Galectin-1 is highly expressed in numerous cancers, such as lung cancer [9], breast cancer [10], pancreatic cancer [11], and ovarian cancer [12]. Galectin-1 overexpression promotes cisplatin resistance in ovarian cancer cells and doxorubicin resistance in triple-negative breast cancer cells [13, 14], indicating the association of Galectin-1 signal transduction with cancer drug resistance. In addition, Galectin-1 expression is positively correlated with advanced lymph node metastasis and poor survival in patients with HCC [15–17]. Receptor tyrosine kinases (RTKs) are involved in various biological functions, such as cell migration, cell invasion, metastasis, and cell cycle regulation. Studies have identified that RTKs are a major contributor to drug resistance in cancer cells and Galectin-1 mediates RTK activation to induce HCC progression [17–20]. However, whether RTK is involved in Galectin-1-mediated sorafenib resistance in HCC remains unclear.

Ferroptosis is a new type of cell death caused by accumulating a large amount of iron and lipid peroxidation [21]. Ferroptosis occurs through two pathways. The first pathway involves the inhibiting system Xc (cystine/glutamate antiporter system), which indirectly inhibits glutathione peroxidase 4 (GPX4), resulting in the accumulation of lipid reactive oxygen species (ROS) and ferroptotic cell death. The second pathway involves iron metabolism. Excessive iron levels contribute to ferroptosis by producing ROS through the Fenton reaction [22]. Accumulating evidence indicates that ferroptosis is centrally involved in tumor growth and chemotherapy sensitivity [23, 24]. For example, the knockdown of long noncoding RNA LINC01134 suppresses GPX4 expression, augmenting oxaliplatin-induced ferroptosis in hepatocarcinoma [25]. Oxaliplatin causes ferroptosis in colorectal cancer cells by inhibiting Nrf2 signaling [26]. Sorafenib is a specific activator of ferroptosis and leads to the accumulation of a large number of iron ions and lipid peroxidation in different cancer types [27]. Suppression of Slc7a11 expression inhibits GPX4 activity, causing lipid peroxidation and triggering sorafenib-induced cardiotoxicity [28]. In addition, SLC27A5 enhances sorafenib-induced ferroptosis in HCC cells, enabling them to overcome sorafenib resistance [29]. Determining molecular mechanisms underlying sorafenib-mediated ferroptosis in HCC may enable the development of a strategy for treating sorafenib resistance.

In this study, we determined whether Galectin-1 expression is correlated with sorafenib resistance in HCC cells. Our results indicate that the inhibition of Galectin-1 expression and the resulting suppression of ferroptosis marker expression (i.e., GPX4 and ferritin heavy chain 1 (FTH1)) led to excessive lipid peroxidation and triggered ferroptosis. In addition, Galectin-1 increased AXL receptor tyrosine kinase (AXL) and proto-oncogene receptor tyrosine kinase (MET) phosphorylation, which induced sorafenib resistance and affected sorafenib-mediated ferroptosis in HCC cells. The present study demonstrates that Galectin-1 induces sorafenib resistance in HCC cells through MET and AXL signaling and reveals a new link between Galectin-1 signaling and sorafenib-mediated ferroptosis.

## RESULTS

### Galectin-1 expression is associated with sorafenib response in HCC cells

**Figure 1:**
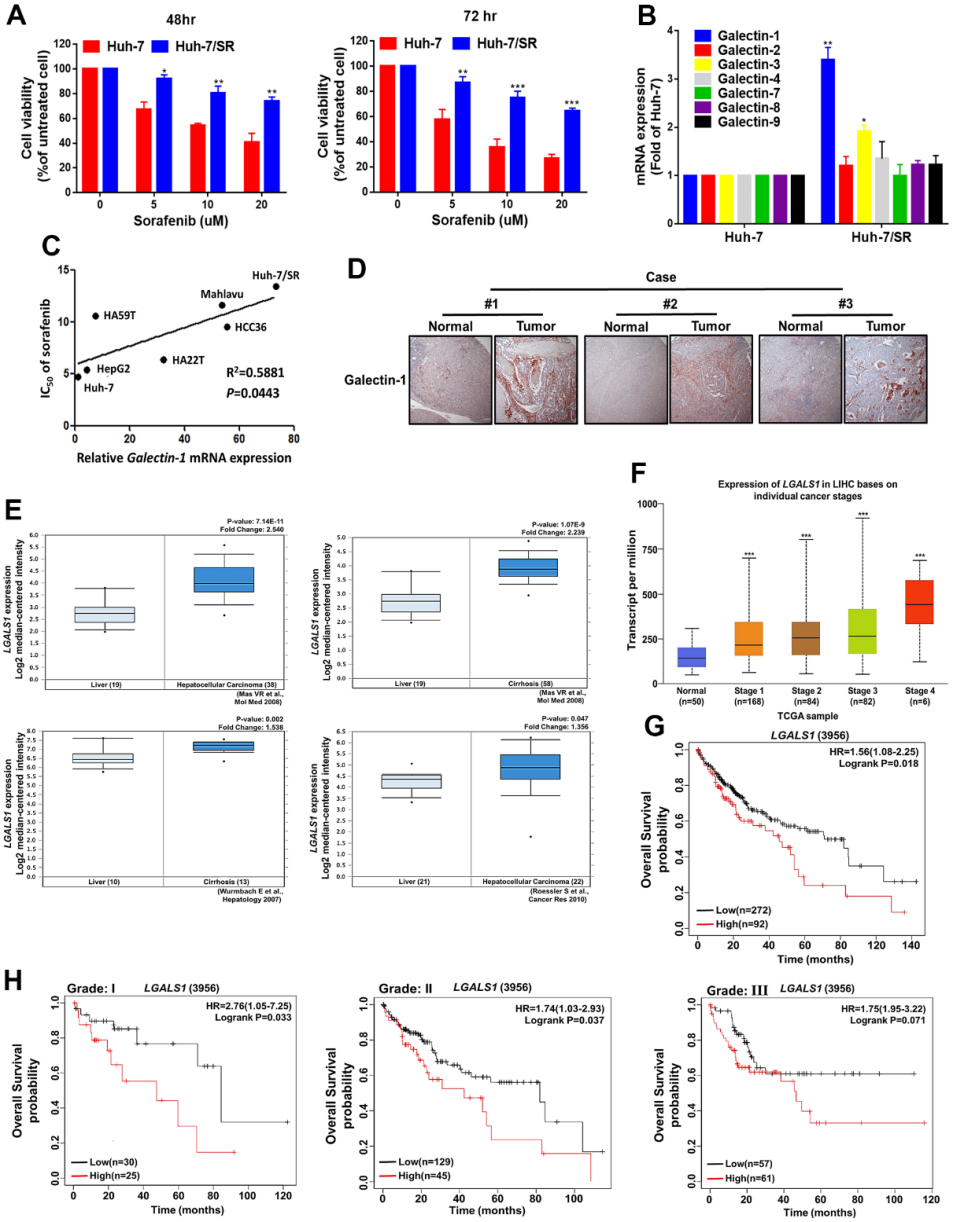
**Galectin-1 expression is associated with patient survival and sorafenib response in HCC cells.** (**A**) Sorafenib-resistant Huh-7 (Huh-7/SR) cells were established and exposed to various sorafenib doses for 48 and 72 h, and cell viability was measured using the 3-(4,5-dimethylthiazol-2-yl)-2,5-diphenyltetrazolium bromide (MTT) assay. (**B**) The mRNA expression of the Galectin family was examined in Huh-7 and Huh-7/SR cells through qRT-PCR. (**C**) Correlation between Galectin-1 expression and IC_50_ of sorafenib in a panel of HCC cells. Pearson’s correlation *r* = 0.5881, and *P* = 0.0443. (**D**) Immunohistochemistry staining was performed to examine Galectin-1 expression in normal liver tissue and HCC tissues. (**E**) The Oncomine database was analyzed to evaluate Galectin-1 expression in HCC tissues compared with normal tissues, with statistics from individual studies obtained from the Oncomine database; fold change (Log2 median-centered intensity) and *P* values are presented within the box plot. (**F**) Evaluation of Galectin-1 expression in different HCC stages by using The Cancer Genome Atlas database (http://ualcan.path.uab.edu/index.html). (**G**) Kaplan–Meier plot revealing the association of Galectin-1 with overall survival in patients with HCC. High Galectin-1 expression was associated with poor survival in patients with HCC. Hazard ratios and *P* values are presented within the box plot. (**H**) Analysis of HCC cells of different tumor grades revealed that high Galectin-1 expression was associated with low overall survival. Hazard ratios and *P* values are presented within the box plot. Data are representative of at least three independent experiments performed in triplicate. Data are presented as means ± standard deviations. *P < 0.05, **P < 0.01, and ***P < 0.001 (Student’s *t* test).

To elucidate the molecular mechanisms underlying sorafenib-mediated drug resistance, we established a sorafenib-resistant HCC cell line (Huh-7/SR) by subjecting Huh-7 cells to stepwise increases in the sorafenib concentration in the culture medium. We examined resistance by using the 3-(4, 5-dimethylthiazol-2-yl)-2, 5-diphenyltetrazolium bromide (MTT) assay. The results indicate that the viability of Huh-7/SR cells was higher than that of parental Huh-7 cells under different sorafenib concentrations (Figure 1A). The galectin family (Galectin-1, -2, -3, -4, -7, -8, and -9) may be associated with cancer progression [30–32]. We compared falectin-1, -2, -3, -4, -7, -8, and -9 mRNA and protein expression levels in Huh-7 and Huh-7/SR cells through quantitative real-time polymerase chain reaction (qRT-PCR) and Western blotting. The mRNA and protein expression of Galectin-1 but not Galectin-2, -3, -4, -7, -8, and -9 were higher in the Huh-7/SR cells (Figure 1B and Supplementary Figure 1). We also analyzed whether Galectin-1 expression is associated with sorafenib sensitivity in different HCC cells (Huh-7, HepG2, HA59T, HA22T, HCC36, Mahlavu, and Huh-7/SR). We observed that Galectin-1 mRNA expression was positively associated with sorafenib resistance in HCC cells (Figure 1C). These findings indicate that Galectin-1 is positively associated with sorafenib resistance and HCC progression. To verify the clinical relevance of our results, we analyzed the association of Galectin-1 with the clinical parameters of patients with liver cancer and observed that Galectin-1 expression was markedly upregulated in liver tissues compared with normal tissues. We examined the Oncomine database and determined that Galectin-1 expression in liver tissues was significantly higher in patients with HCC or cirrhosis than in those without HCC or cirrhosis (Figure 1E). In addition, an analysis of The Cancer Genome Atlas (TCGA) database revealed that Galectin-1 expression was positively correlated with HCC stage (Figure 1F). The overall survival rate of the high Galectin-1 expression group was significantly lower than that of the low Galectin-1 expression group (Figure 1G). In addition, we analyzed the correlation between Galectin-1 expression and overall survival in patients with different HCC tumor grades. The overall survival rate of the high–Galectin-1 expression group was markedly lower than that of the low–Galectin-1 expression group across different HCC tumor grades (Figure 1H). These results indicate that high Galectin-1 expression is correlated with sorafenib resistance and poor prognosis in patients with HCC.

### Enhancement of sorafenib resistance and suppression of sorafenib-mediated ferroptosis in HCC cells by Galectin-1 overexpression

**Figure 2:**
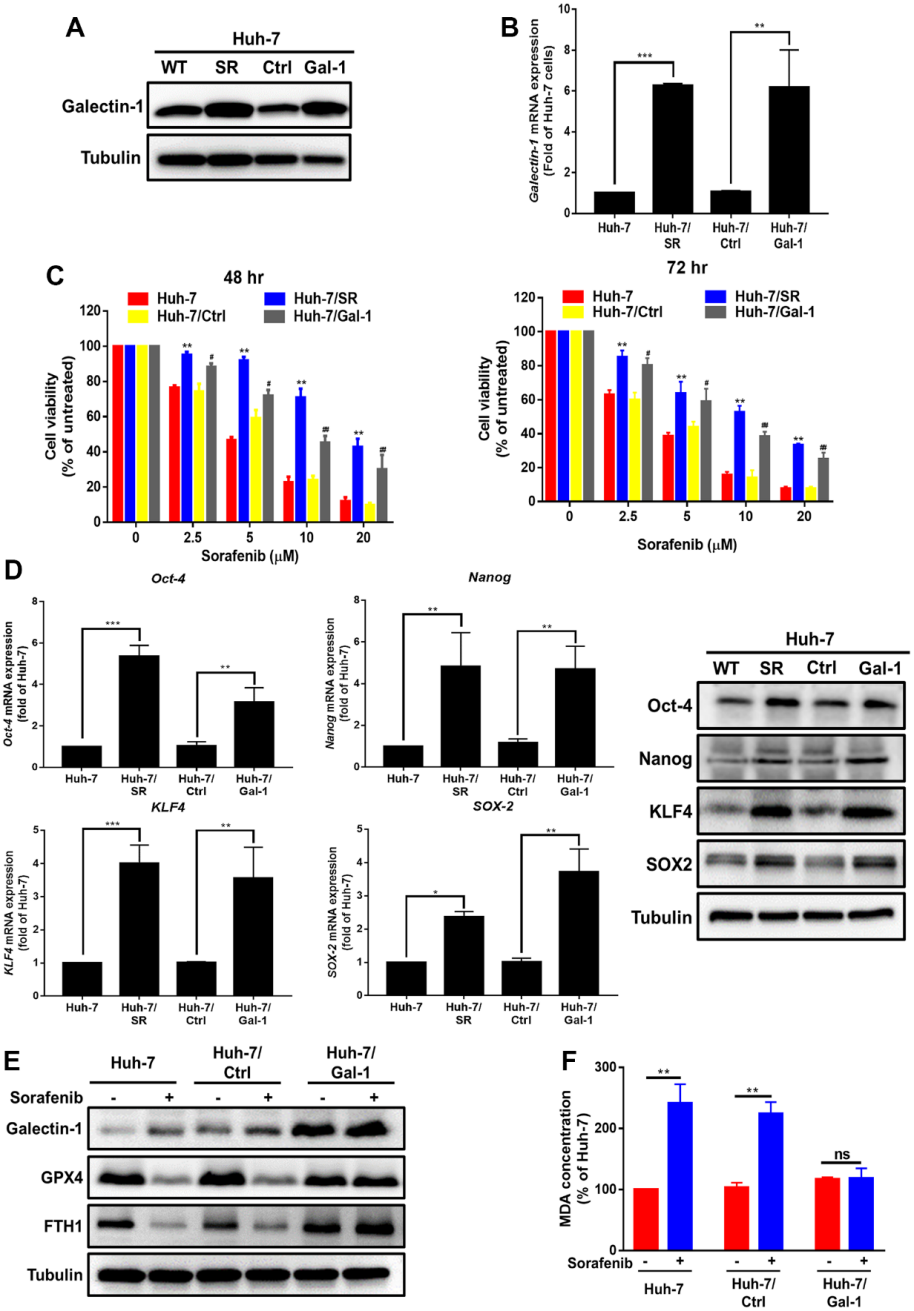
**Enhanced sorafenib resistance and suppressed sorafenib-mediated ferroptosis in HCC cells with Galectin-1 overexpression.** (**A**, **B**) Analysis of Galectin-1 expression in Huh-7, Huh-7/SR, vector control (Huh-7/Ctrl), and Galectin-1 overexpressing Huh-7 (Huh-7/Gal-1) cells through Western blotting and qRT-PCR. (**C**) Cell viability of HCC cells treated with various doses of sorafenib was examined using the MTT assay. (**D**) Analysis of cancer stem cell marker (*Oct-4*, *Nanog*, *SOX-2*, and *KLF4*) expression through qRT-PCR and Western blotting. (**E**) Detection of Galectin-1, glutathione peroxidase 4 (GPX4), and FTH-1 expression in Galectin-1-overexpressing cells, cells treated with sorafenib (10 μM) for 48 h, and cells not treated with sorafenib. (**F**) Lipid peroxidation detected using the malondialdehyde (MDA) assay. Data are presented as means ± standard deviations. *P < 0.05, **P < 0.01, and ***P < 0.001 (Student’s *t* test).

To investigate the effect of Galectin-1 on sorafenib sensitivity and cancer progression in HCC cells, plasmids with Galectin-1 overexpression were transfected into parental Huh-7 cells. Galectin-1 overexpression was verified through Western blotting (Figure 2A) and qRT-PCR (Figure 2B). We performed the MTT assay to determine the effect of Galectin-1 on sorafenib sensitivity. The results demonstrated that Huh-7 cells with Galectin-1 overexpression (Huh-7/Gal) exhibited a significantly lower sorafenib sensitivity than did control-transfected cells (Huh-7/Ctrl; Figure 2C) at 48 and 72 h. We used HepG2 cells to confirm these results and observed that Galectin-1 overexpression in HepG2 cells (HepG2/Gal) significantly increased Galectin-1 mRNA and protein expression (Supplementary Figure 2A, 2B) and enhanced their sorafenib resistance (Supplementary Figure 2C). Cancer stem cells have been linked to therapeutic resistance and tumor recurrence [33, 34]. For this reason, we presumed that Galectin-1 would regulate the cancer stemness of HCC. The results of qRT-PCR and Western blotting indicate that Galectin-1 overexpression increased the mRNA and protein expression of associated stemness-related markers (Oct-4, Nanog, SOX-2, and KLF4; Figure 2D). These data demonstrate that Galectin-1 overexpression reduced sorafenib sensitivity and cancer stemness in HCC cells. Accumulating evidence indicates that sorafenib induces ferroptosis in HCC cells [35–37]. We hypothesized that sorafenib-mediated ferroptosis in HCC would be regulated by Galectin-1 expression. To verify this hypothesis, we examined the expression of the ferroptosis markers GPX4 and FTH1 through Western blotting and discovered that sorafenib reduced GPX4 and FTH1 expression in Huh-7 cells. Subsequently, Galectin-1 overexpression recovered the expression of GPX4 and FTH1 and abolished sorafenib-mediated ferroptosis (Figure 2E). To verify these results, we performed a lipid peroxidation (MDA) assay and determined that Galectin-1 overexpression abolished sorafenib-induced lipid peroxidation (Figure 2F). These data indicate that Galectin-1 overexpression promotes sorafenib resistance and reduces sorafenib-mediated ferroptosis in HCC cells.

### Loss of Galectin-1 restores sorafenib sensitivity and ferroptosis activation in HCC cells

**Figure 3:**
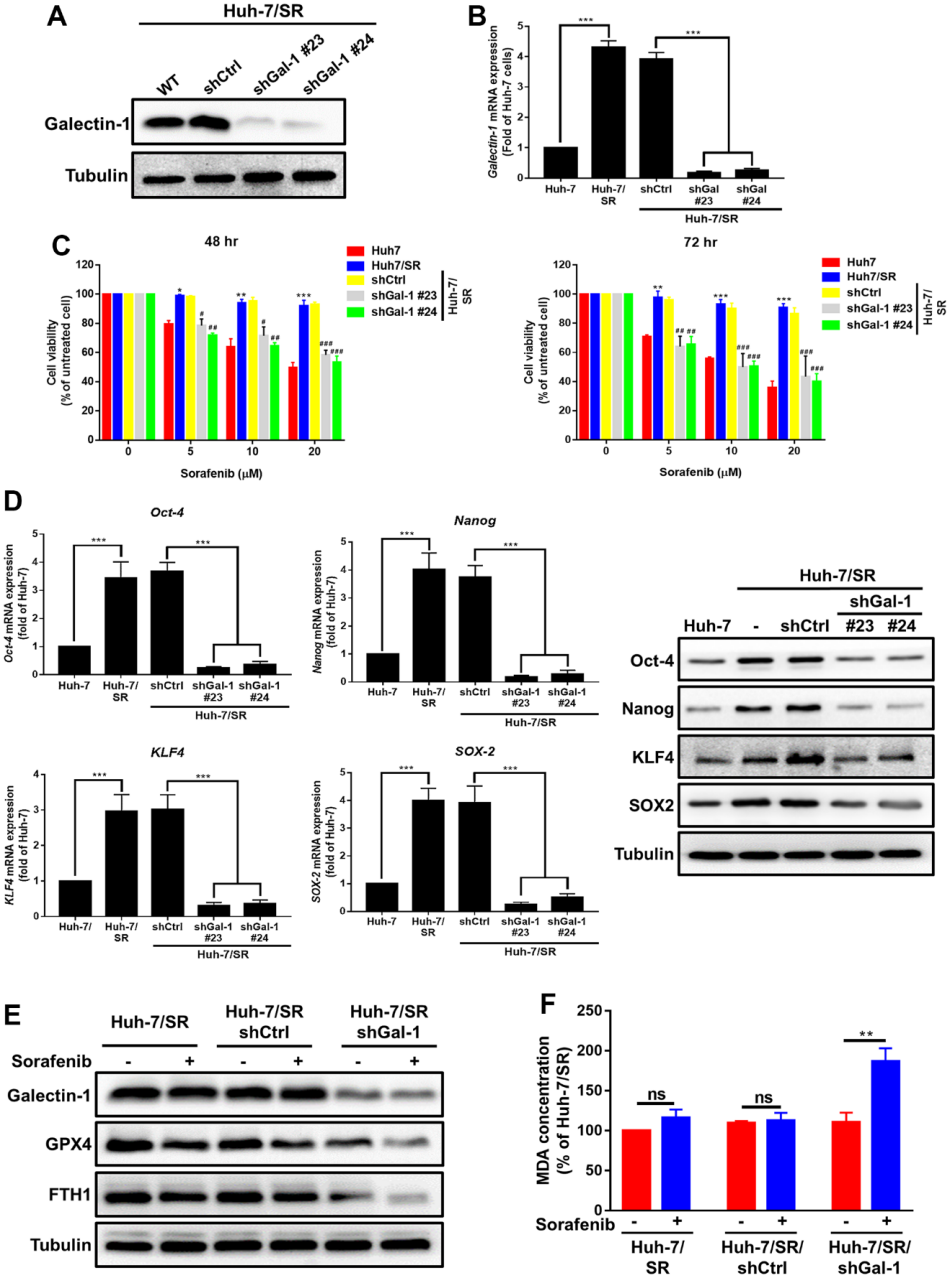
**Loss of Galectin-1 overcomes sorafenib sensitivity and promotes ferroptosis in HCC cells.** (**A**, **B**) Stable Galectin-1-silenced Huh-7/SR (Huh-7/SR/shGal#23 and #24) and control (Huh-7/SR/shCtrl) cells were analyzed using Western blotting and qRT-PCR. (**C**) Sorafenib sensitivity in indicated cells analyzed for 48 and 72 h by using the MTT assay. (**D**) qRT-PCR and Western blotting were used to determine the expression of cancer stem cells markers (*Oct-4*, *Nanog*, *SOX-2*, and *KLF4*). (**E**) Western blotting analysis of Galectin-1, GPX4, and FTH-1 expression in Galectin-1-knockdown sorafenib-resistant HCC cells, cells treated with sorafenib (10 μM) for 48 h, and cells not treated with sorafenib, respectively. (**F**) Lipid peroxidation determined using the MDA assay. Data are presented as means ± standard deviations. *P < 0.05 and **P < 0.01 for Huh-7; #*P* < 0.05, ##*P* < 0.01, and ###*P* < 0.001 for Huh-7/SR/shCtrl (Student’s *t* test).

To determine whether Galectin-1 mediates sorafenib sensitivity and ferroptosis activation in HCC cells, we conducted a loss-of-function experiment to verify whether Galectin-1 mediates sorafenib resistance in HCC cells. We transfected Galectin-1 knockdown plasmids into sorafenib-resistant Huh-7/SR cells and examined the transfection efficiency through qRT-PCR and Western blotting. Galectin-1 knockdown in Huh-7/SR cells was achieved through the transfection of Galectin-1 knockdown plasmids (Huh-7/SR/shGal-1 #23 and Huh-7/SR/shGal-1 #24; Figure 3A, 3B). The MTT assay revealed that sorafenib resistance was lower in Huh-7/SR cells with Galectin-1 knockdown (Huh-7/SR/shGal-1 #23 and Huh-7/SR/shGal-1 #24 cells) than in cells without Galectin-1 knockdown (Figure 3C). We performed the same analysis by using Mahlavu cells. The results indicate that Galectin-1 knockdown in Mahlavu cells reduced resistance to sorafenib (Supplementary Figure 3A, 3B). The inhibition of Galectin-1 in Huh-7/SR cells reduced Oct-4, Nanog, SOX-2, and KLF4 protein and mRNA expression (Figure 3D). These results demonstrate that the knockdown of Galectin-1 expression reversed sorafenib resistance and reduced cancer stemness in Huh-7/SR cells. We validated the role of Galectin-1 in sorafenib-mediated ferroptosis and observed that Galectin-1 underexpression inhibited GPX4 and FTH1 expression after sorafenib treatment (Figure 3E). We also examined lipid peroxidation in Huh-7/SR/shGalectin-1 cells and observed a substantial degree of lipid peroxidation in sorafenib-resistant cells with Galectin-1 knockdown (Figure 3F). Collectively, our data demonstrate that the knockdown of Galectin-1 reversed sorafenib resistance, reduced cancer stemness, and enhanced sorafenib-mediated ferroptosis.

### Involvement of AXL and MET signaling in Galectin-1-mediated sorafenib resistance and ferroptosis in HCC

**Figure 4:**
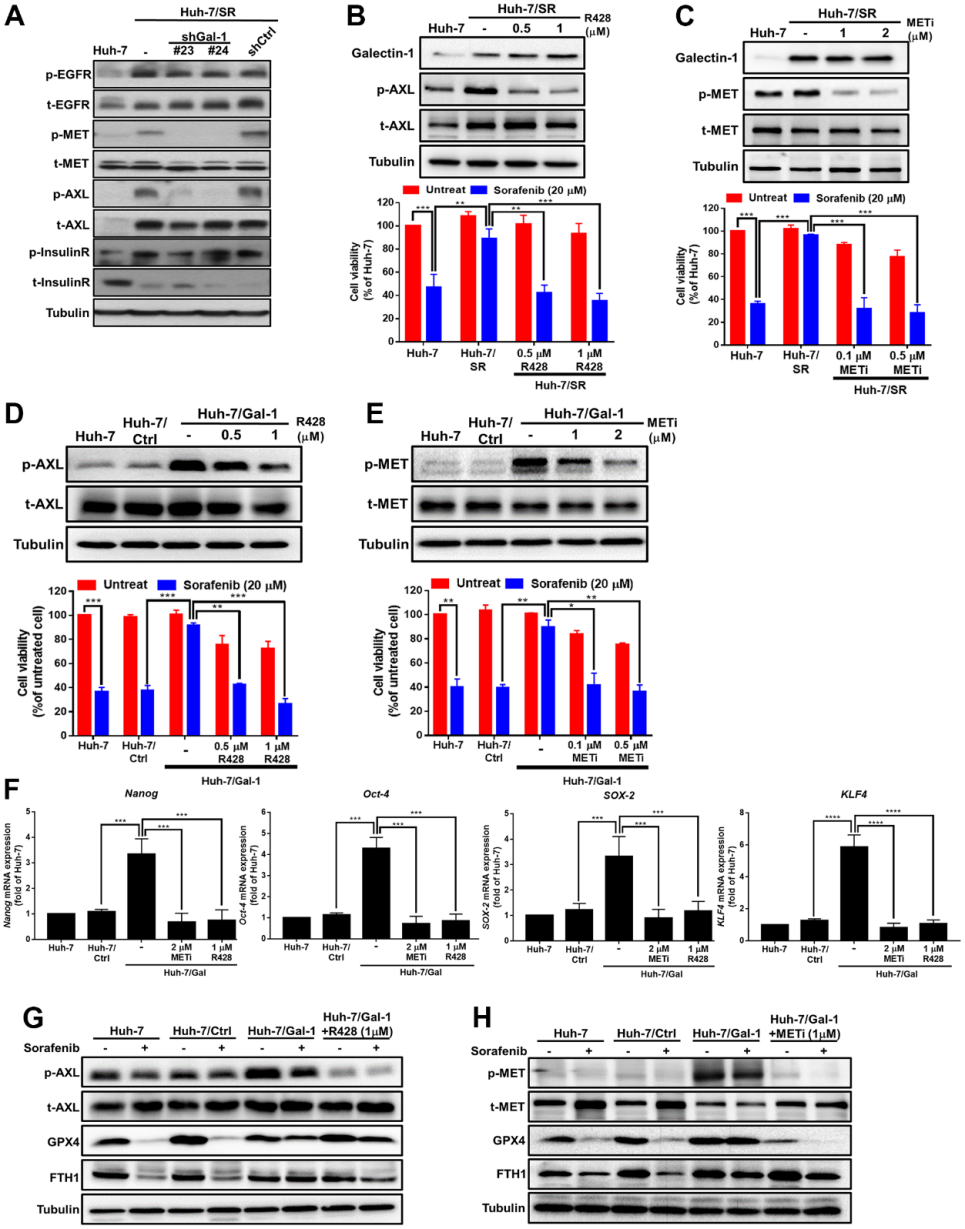
**MET and AXL signaling involved in Galectin-1-mediated sorafenib resistance and ferroptosis in HCC.** (**A**) Galectin-1-knocked-down Huh-7/SR (Huh-7/SR/shGal#23 and #24) and control (Huh-7/SR/shCtrl) cells were analyzed for RTK expression (EGFR, MET, AXL, and insulin receptor) through Western blotting. Huh-7/SR cells were treated with an MET inhibitor and AXL inhibitor R428 for 48 hr. Analysis of Galectin-1, AXL, and phospho-AXL expression (**B** upper panel) and MET and phospho-MET expression (**C** upper panel) were performed using Western blotting. Cell viability of Huh-7/SR cells cotreated with 20 μM of sorafenib and AXL (**B** lower panel) or an MET inhibitor (**C** lower panel) for 48 h. Galectin-1 overexpression after treatment with the MET inhibitor and AXL inhibitor R428 for 48 h. Analysis of AXL and phospho-AXL expression (**D** upper panel) and MET and phospho-MET expression (**E** upper panel) by using Western blotting. Cell viability of Galectin-1- overexpressing cells cotreated with 20 μM of sorafenib and AXL (**D** lower panel) or a MET inhibitor (**E** lower panel) for 48 h. (**F**) Oct-4, Nanog, SOX-2, and KLF4 mRNA expression determined using qRT-PCR in Galectin-1-overexpressing cells treated with an MET inhibitor and an AXL inhibitor R428. Western blot analysis was used to detect AXL, phospho-AXL, GPX4, and ferritin heavy chain 1 expression in cells cotreated with 20 μM of sorafenib and an (**G**) AXL inhibitor or (**H**) MET inhibitor. Data are presented as means ± standard deviations. **P < 0.01, and ***P < 0.001 (Student’s *t* test).

RTKs play a crucial role in drug resistance, and sorafenib is a small-molecule tyrosine kinase inhibitor. Some studies have suggested that epidermal growth factor receptor (EGFR), MET, AXL, and InsulinR are involved in drug resistance mechanisms [38–41]. We hypothesized that RTKs would be involved in the Galectin-1-mediated sensitization of HCC cells to sorafenib. These candidate RTKs were validated, and their differential expression was confirmed through Western blotting. We determined that sorafenib-resistant HCC cells exhibited increased EGFR, MET, AXL, and InsulinR phosphorylation. MET and AXL phosphorylation were significantly lower in Huh-7/SR/shGal-1 cells than in scramble cells, whereas EGFR and InsulinR phosphorylation did not considerably differ between Galectin-1 knockdown Huh7/SR cells and scramble cells (Figure 4A). These results indicate the involvement of AXL and MET in Galectin-1-mediated sorafenib resistance. To investigate whether AXL and MET are involved in the Galectin-1-mediated sorafenib sensitization of HCC cells, we treated Huh-7/SR cells with the AXL inhibitor R428 and the MET inhibitor GSK1363089 and analyzed AXL, MET, and Galectin-1 protein expression through Western blotting. We discovered that treatment with AXL and MET inhibitors successfully inhibited AXL and MET phosphorylation, respectively, but did not affect Galectin-1 expression (Figure 4B, 4C, upper panel). We performed the MTT assay to investigate whether treatment with AXL and MET inhibitors affects the sorafenib-mediated viability of Huh-7/SR cells (Figure 4B, 4C, lower panel). The results indicate that the inhibition of AXL and MET phosphorylation significantly increased the sorafenib sensitivity of Huh-7/SR cells. We next investigated whether AXL and MET are involved in the Galectin-1-mediated sorafenib sensitization of HCC cells. The overexpression of Galectin-1 in Huh-7 cells upregulated the expression of phosphorylated AXL and MET proteins, and treatment with AXL and MET inhibitors reduced the phosphorylation of AXL and MET (Figure 4D, 4E, upper panel). The MTT assay was used to determine whether AXL and MET inhibitors affect Galectin-1-mediated sorafenib resistance. The results demonstrate that the overexpression of Galectin-1 significantly reduced sorafenib sensitivity, whereas the inhibition of AXL and MET phosphorylation reduced resistance to sorafenib (Figure 4D, 4E, lower panel). In addition to using AXL and MET inhibitors, we knocked down AXL and MET. The results indicate that AXL and MET knockdown restored the sorafenib sensitivity of Huh-7 cells under Galectin-1 overexpression (Supplementary Figure 4A, 4B). Treatment with AXL and MET inhibitors successfully abolished Galectin-1 overexpression–mediated cancer stemness (Figure 4F). We investigated the effect of AXL and MET inhibition on the role of Galectin-1 in mediating sorafenib sensitivity and sorafenib-mediated ferroptosis. Western blot analysis revealed that GPX4 and FTH1 expression was lower in Galectin-1-overexpressing cells after cotreatment with an AXL inhibitor (Figure 4G) or an MET inhibitor (Figure 4H) and sorafenib. These results indicate the involvement of AXL and MET in Galectin-1-mediated sorafenib resistance and sorafenib-mediated ferroptosis in HCC cells.

### Clinical significance of AXL, MET, and Galectin-1 in patients with HCC

**Figure 5:**
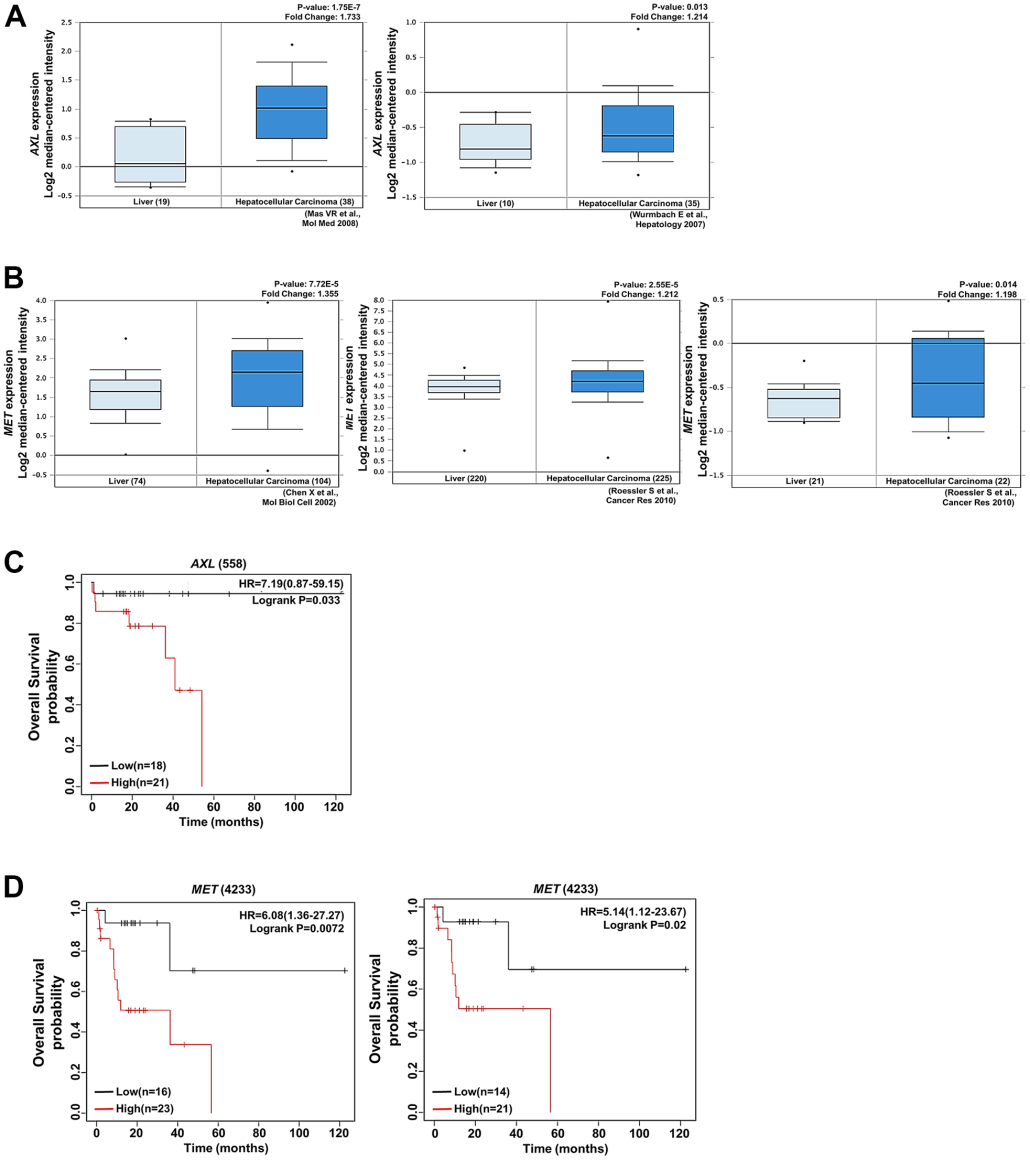
**AXL and MET expression was positively associated with HCC.** (**A**) AXL expression was positively correlated with HCC tissues and cirrhosis in the analysis of the Oncomine database (https://www.oncomine.com/). (**B**) MET expression was significantly upregulated in HCC tissues compared with normal tissues according to the analysis of data in the Oncomine database. (**C**) Kaplan–Meier plot presenting the association of AXL with HCC. High expression of AXL was associated with poor overall survival in patients with HCC. (**D**) Kaplan–Meier plot presenting the association of MET with overall survival in patients with HCC. High expression of MET was associated with poor progression-free survival and relapse-free survival in patients with HCC.

Our findings indicate that Galectin-1 regulated sorafenib resistance in HCC cells through AXL and MET activation. We used the Oncomine database (https://www.oncomine.com/) to determine the clinical associations of AXL, MET, and Galectin-1 with HCC. We observed that the expression of AXL and MET was higher in HCC cells than in normal tissues (Figure 5A, 5B). High MET expression was associated with poor progression-free and relapse-free survival (Figure 5C). Similarly, high AXL expression was associated with poor overall survival in patients with HCC (Figure 5D). These results indicate that high AXL and MET expression was associated with poor prognosis in patients with HCC. We analyzed the TCGA database and observed that AXL expression was positively associated with Galectin-1 expression (Supplementary Figure 5A). The overall survival rates were lower in the group with Galectin-1 and MET overexpression (Supplementary Figure 5B) and in the group with Galectin-1 and AXL overexpression (Supplementary Figure 5C) than in groups without Galectin-1 and AXL or MET overexpression. These results indicate that Galectin-1 expression is positively associated with AXL and MET signaling; this contributes to sorafenib resistance in HCC cells.

## DISCUSSION

Sorafenib is the first targeted drug clinically approved for the treatment of advanced-stage HCC. Sorafenib targets the Raf/mitogen-activated protein kinase/extracellular signaling–regulated kinase pathway and various RTKs, including EGFR, vascular endothelial growth factor receptor, platelet-derived growth factor receptor, FMS-like tyrosine kinase-3, and c-kit [42–44]. We established a sorafenib-resistant HCC cell line, Huh-7/SR, to examine molecular mechanisms underlying sorafenib resistance. We determined that Huh-7/SR cells had a higher level of Galectin-1 expression than did parental Huh-7 cells and that Galectin-1 expression was inversely related to the sorafenib response in HCC cell lines. Galectin-1 knockdown in Huh-7/SR cells significantly reduced MET and AXL phosphorylation, which was positively associated with the sorafenib response in HCC. Taken together, these results indicate that Galectin-1 plays a critical role in activating MET/AXL signaling and enhancing sorafenib resistance in HCC.

Galectins are a family of beta-galactoside-binding proteins, and a growing body of evidence has indicated that galectins promote cancer progression by enhancing oncogenic signaling pathways, modulating tumor cell growth or apoptosis, regulating cell migration and invasion, and mediating drug resistance [30, 31, 45, 46]. A high Galectin-1 level promotes pancreatic cancer cell proliferation and tumor metastasis [11, 47]. In addition, increased galectin-3 expression is involved in the liver metastasis of colorectal cancer and promotes breast cancer metastasis [48–50]. Here, we analyzed the mRNA expression of galectin family proteins in Huh-7 and Huh-7/SR cell lines through qRT-PCR. We observed that Galectin-1 mRNA expression was upregulated in Huh-7/SR cells. Therefore, Galectin-1 affects sorafenib resistance in HCC cells. In another study, Galectin-1 overexpression in HCC cells contributed to tumor growth *in vivo* [51]. In addition, Galectin-1 triggers epithelial–mesenchymal transition in HCC cells [20]. Similar results were observed in the context of oral cancer; Galectin-1 regulated matrix metalloproteinase (MMP)-2 and MMP-9 expression, causing tumor invasion [52]. Galectin-1 may drive T-cell exclusion and promote immunotherapy resistance in head and neck cancer [53]. The direct interaction of Galectin-1 with integrin β1 causes resistance to doxorubicin in breast cancer cells [14]. The knockdown of Galectin-1 enhances cisplatin sensitivity in neuroblastoma cells [54]. The results of the present study indicate that Galectin-1 overexpression promotes sorafenib resistance in HCC cells. In addition, Galectin-1 knockdown in Huh-7/SR cells reduces their sorafenib resistance. High Galectin-1 expression has been associated with poor prognosis in various cancers, including ovarian cancer [12], glioblastoma [55], renal cell cancer [56], head and neck squamous cell carcinoma [57], and non–small-cell lung cancer [9]. These results are consistent with our data indicating that Galectin-1 is highly expressed in HCC and that its overexpression reduces overall survival.

Ferroptosis involves iron accumulation and is a lipid peroxidation–dependent type of cell death that is morphologically, biochemically, and genetically distinct from apoptosis, necrosis, autophagy, and other forms of cell death [22]. Ferroptosis plays an essential role in cancer progression and drug resistance in different cancer types. For example, the inhibition of MEX3A expression results in excessive ROS production and lipid peroxidation, thereby enhancing ferroptosis in ovarian cancer [58]. In addition, silencing SLC7A11 expression reduces ROS and MDA accumulation, resulting in ferroptosis in non–small-cell lung cancer [59]. Sorafenib is a potent inducer of ferroptosis, and this mechanism is linked to the efficacy of sorafenib in treating HCC. The suppression of GSTZ1 reduces cell viability and enhances sorafenib-mediated ferroptosis in HCC cells by regulating the NRF2/GPX4 axis [60]. ABCC5 depletion attenuates sorafenib-mediated excessive lipid peroxidation and mitochondrial membrane potential reduction in HCC cells [61]. The findings of the present study demonstrate that the upregulation of Galectin-1 exacerbates resistance to sorafenib-mediated ferroptosis by reducing excessive lipid peroxidation in sorafenib-sensitive HCC cells. By contrast, Galectin-1 underexpression inhibits the expression of the sorafenib-mediated ferroptosis markers GPX4 and FTH1 and increases lipid peroxidation, restoring the sensitivity to sorafenib of sorafenib-resistant HCC cells.

Several studies have reported on the relationship between stemness and iron stability, leading to a new term called FEROSTEM (“FER” for iron and “STEM” for stem cells) [62]. Glioblastoma cancer stem cells expressed a higher level of transferrin (receptor transferrin receptor 1) than did nonglioblastoma cancer stem cells [63]. Iron uptake and chelator-induced cell death were enhanced in an *in vitro* cancer stem cell tumorsphere model of breast cancer compared with a monolayer cell culture [64]. The inhibition of SLC7A11 reduced chemoresistance and stemness in colon cancer and gastric cancer cells by triggering ferroptosis [65]. Lung cancer stem-like cells exhibited higher SLC7A11 expression that did lung cancer cells, and the stem cell transcription factor SOX2 activated SLC7A11, thus enhancing the resistance of lung cancer stem-like cells to ferroptosis [66]. In addition, the suppression of FTH expression regulated the expression of specific cancer stem cell markers and spheroid formation in other solid tumor types [63]. Esophageal cancer stem-like cells exhibited higher GPX4 and xCT expression, escaping ferroptosis-induced cell death [67]. Therefore, ferroptosis may be a weakness of cancer stem cells and can be exploited therapeutically to target cell death more effectively in anticancer stem cell therapies.

RTKs are cell surface receptors involved in signaling transduction that occurs in response to various ligands, such as growth factors, cytokines, and hormones [68]. RTKs are involved in various biological functions, such as cell migration, cell invasion, metastasis, cell cycle regulation, stem cell features, and resistance to anticancer drugs [69, 70]. Several studies have demonstrated that RTKs are overexpressed in HCC, lung cancer, gastric cancer, colorectal cancer, pancreatic cancer, breast cancer, ovarian cancer, prostate cancer, and glioma [71–74]. Other studies have observed an association of RTK dysfunction with alterations in chemotherapy sensitization. In breast cancer, RTKs regulate the PI3K/Akt and Ras/Raf/mitogen-activated protein kinase pathways and thereby confer trastuzumab resistance [75]. In addition, RTKs regulate E1A through NFI-C and NFI-X inhibition and AXL expression and thus sensitize breast cancer cells to EGFR–tyrosine kinase inhibitor chemotherapy [76]. A study indicated that EGFR TKIs blocked Akt and Ets-1 activity and thus evoked gefitinib resistance in non–small-cell lung cancer cells [77]. Galectins can mediate RTK activation and induce the progression of various cancers [78]. In the present study, we determined that EGFR, MET, AXL, ephrin receptor, and insulin receptor phosphorylation were significantly higher in Huh-7/SR cells than in parent Huh-7 cells. We demonstrated that the downregulation of Galectin-1 inhibited phospho-MET and phospho-AXL expression. Treatment with MET and AXL inhibitors in Huh-7/SR cells overexpressing Galectin-1 significantly increased the sensitivity of these cells to sorafenib, indicating that Galectin-1 enhances MET/AXL signaling and contributes to sorafenib resistance in HCC cells.

**Figure 6:**
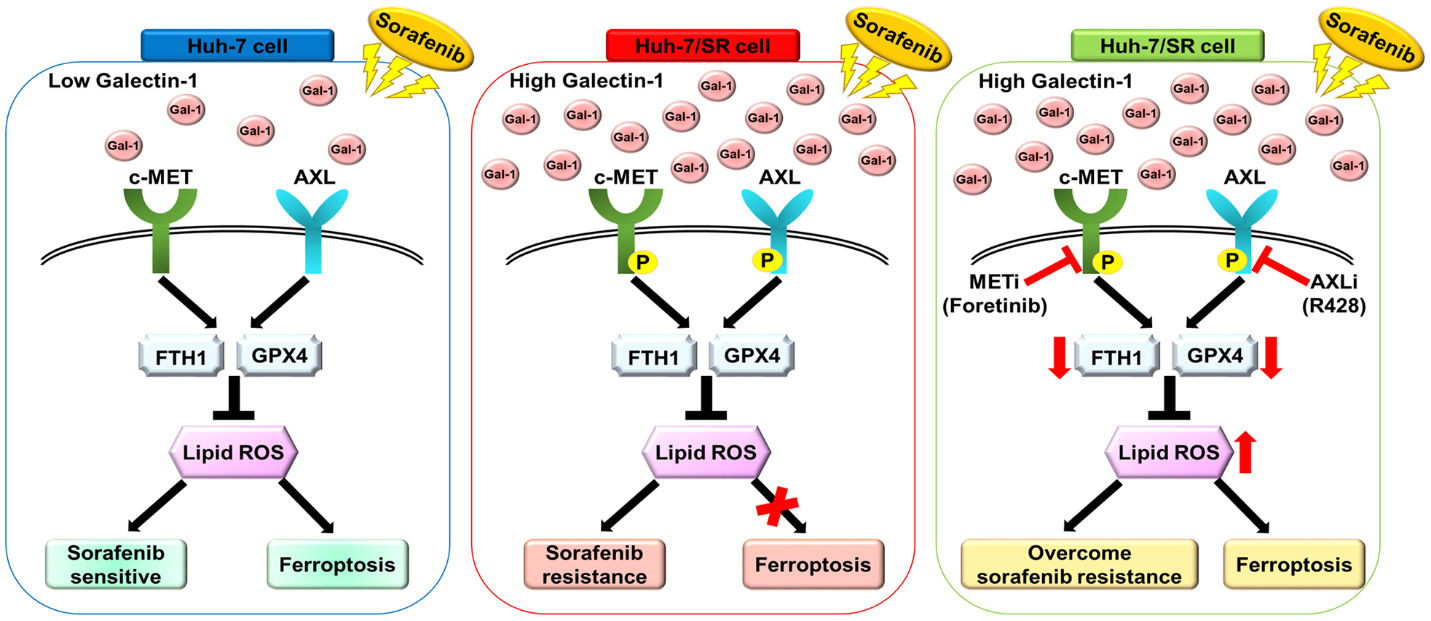
**Schematic for Galectin-1-mediated AXL/MET signaling in sorafenib resistance in HCC cells.** Galectin-1 was significantly overexpressed in Huh-7/SR cells and promoted MET and AXL phosphorylation, contributing to sorafenib resistance and decreased sorafenib-mediated ferroptosis. Combined treatment with sorafenib and the AXL/MET inhibitor blocked Galectin-1-mediated AXL/MET signaling, overcoming sorafenib resistance and sorafenib-mediated ferroptosis in Huh-7/SR cells.

To the best of our knowledge, this is the first study to examine the relationship between Galectin-1-mediated sorafenib resistance and sorafenib-mediated ferroptosis in HCC. In the present study, we demonstrated that Galectin-1 overexpression induced resistance to sorafenib and inhibited sorafenib-mediated ferroptosis. By contrast, Galectin-1 knockdown restored sorafenib resistance and sorafenib-mediated ferroptosis. Our results indicate that Galectin-1 enhances resistance to sorafenib-mediated ferroptosis by regulating MET/AXL signaling in HCC cells (Figure 6A). Additionally, we observed that a high Galectin-1 level was associated with AXL expression and poor overall survival outcomes in patients with HCC. When Galectin-1 and downstream MET/AXL signaling were combined, the overall survival rate was lower in the group with high expression. Our study has some limitations that should be addressed. First, we did not determine the correlation between Galectin-1 and MET in HCC clinical databases. Studies should analyze a larger cohort to confirm our results. Second, we did not clarify how Galectin-1 regulates GPX4 and FTH-1, causing ferroptosis in sorafenib-resistant HCC cells. We plan to conduct experiments in our next study to elucidate the regulatory mechanism or signal transduction pathway through which Galectin-1 regulates the expression of GPX4 and FTH-1. Our findings indicate that Galectin-1 is a potential biomarker that can be used to determine sorafenib responses and develop new therapeutic strategies to overcome sorafenib resistance in patients with HCC.

## MATERIALS AND METHODS

### Cell culture and sorafenib-resistant cell establishment

Human HCC cell lines Huh-7, HepG2, HA59T, HA22T, HCC36, and Mahlavu and sorafenib-resistant cells (Huh-7/SR) were grown in Dulbecco’s modified Eagle medium (Corning, USA) supplemented with 10% fetal bovine serum and 100 U/mL penicillin–streptomycin–amphotericin B (Biological Industries) and were incubated at 37° C in a humidified atmosphere containing 5% CO_2_. To induce sorafenib resistance, Huh-7 cells were treated with 0.25 μM sorafenib (BAY 43-9006, Selleck Chemicals, USA), and the dose was gradually increased every week for 6 months. The resistant cells were maintained at a final dose of 10 μM.

### MTT assay

A total of 3000 cells per well were seeded in 96-well plates. Viable cells were stained with MTT (Promega). After the removal of the medium, 100 μL of dimethyl sulfoxide was used to dissolve formazan crystals. Absorbance at 570 and 630 nm was measured using a SpectraMax M3 Multi-Mode Microplate Reader (Molecular Devices LLC, USA).

### qRT-PCR assay

Total RNA was extracted from cells using NucleqoZOL (Macherey-Nagel, Germany), and RNA was reversed transcribed to complementary DNA by using a Moloney murine leukemia virus reverse transcription kit (Protech Technology). Subsequently, qRT-PCR was performed using the SYBR Green PCR Master Mix (Applied Biosystems) in the CFX96 Touch Real-Time PCR Detection System (Bio-Rad, USA). Data were analyzed using the relative quantification comparative threshold cycle method with the formula 2 (2 − ΔΔCt). Primer sequence details are listed in Table 1.

**Table 1:** Primer sequences information for qRT-PCR.

**Galectin-1**
GCCTGCCCGGGAACAT (Forward)
CTGGCGACCAGACCACAAG (Reverse)
**Galectin-2**
GCTTCAGCGAATCCACCATT (Forward)
GTTCTTGCCCCCAGTTGCT (Reverse)
**Galectin-3**
CCATTTGAAAGTGGGAAACCA (Forward)
CATCATTCACTGCAACCTTGAAG (Reverse)
**Galectin-4**
CCAGCACCTCTTTGACTTTGC (Forward)
CAATGTGTCCACCCTCTGGAA (Reverse)
**Galectin-7**
CTGGCACGGTGCTGAGAAT (Forward)
GGAACCTGCTGGCATTGG (Reverse)
**Galectin-8**
TGAATGCAAATGCCAAAAGC (Forward)
TGGGTTCAAGTGTAGAGCAATATCC (Reverse)
**Galectin-9**
ATGCTGTGGTCCGCAACA (Forward)
GGCAGACTTCGCTCCTCAGA (Reverse)
**Oct-4**
GCAATTTGCCAAGCTCCTGAA (Forward)
GCAGATGGTCGTTTGGCTGA (Reverse)
**SOX2**
GGGGGAATGGACCTTGTATAG (Forward)
GCAAAGCTCCTACCGTACCA (Reverse)
**Nanog**
AATGGTGTGACGCAGGGATG (Forward)
TGCACCAGGTCTGAGTGTTC (Reverse)
**KLF4**
GGGAGAAGACACTGCGTCA (Forward)
GGAAGCACTGGGGGAAGT (Reverse)
**GAPDH**
AGCCACATCGCTCAGACAC (Forward)
GCCCAATACGACCAAATCC (Reverse)

### Western blot analysis

Total proteins were extracted from cells by using a radioimmunoprecipitation assay lysis buffer with a protease inhibitor cocktail (MedChemExpress, NJ, USA). Equal amounts of whole-cell lysates were used for sodium dodecyl sulfate–polyacrylamide gel electrophoresis, and proteins were electrophoretically transferred onto 0.22-μm polyvinylidene difluoride membranes (Immobilon, Millipore, USA). The membranes were blocked with 5% bovine serum albumin (Bio Basic, Canada) in Tris buffered saline with Tween buffer and subsequently incubated with primary antibodies at 4° C overnight. The membranes were then incubated with a horseradish peroxidase–conjugated antibody (Jackson ImmunoResearch, West Grove, PA, USA) at room temperature for 1 h. The membranes were visualized using a chemiluminescent horseradish peroxidase substrate (Millipore) on a FluorChem FC3 System (Bio-Techne, Minneapolis, MN, USA). The following primary antibodies were used: anti-Galectin-1 (1:1000; ab112525; Abcam), anti-Galectin-2 (1:250; A6645; ABclonal), anti-Galectin-3 (1:1000; A11198; ABclonal), anti-Galectin-4, anti-Galectin-7 (1:100; cs-137085; Santa Cruz), anti-Galectin-8, anti-Galectin-9 (1:1000; bs-1699R, Bioss), anti-KLF4 (1:1000; A13673; ABclonal), anti-SOX2 (1:1000; A9118; ABclonal), anti-OCT-4 (1:500; A7920; ABclonal), anti-Nanog (1:1000; A3232; ABclonal), anti-EGFR (1:1000; #2232; Cell Signaling), anti-phospho-EGFR (Tyr1068; 1:1000; #2234; Cell Signaling), anti-MET (1:1000; A0040; ABclonal), anti-phospho-MET (Tyr1349; 1:500; AP0077; ABclonal), anti-AXL (1:1000; A17874; ABclonal), antiphospho-AXL (Tyr702; 1:500; AP0848; ABclonal), anti-insulin receptor (ab137747; Abcam), antiphospho-insulin receptor (Tyr1361; 1:500; ab60946; Abcam), and anti-α-tubulin (T5168; Sigma-Aldrich).

### Galectin-1 plasmid transfection

Full-length human Galectin-1 (NM_002305) was subcloned into pcDNA6 (Invitrogen). Plasmid pcDNA6 vector-Galectin-1 (pGalectin-1) and empty pcDNA6 vector-plasmid were used as controls. We purchased pLKO.1-puro-based lentiviral vectors from the National RNAi Core Facility (Academia Sinica, Taipei, Taiwan) for lentiviral production. Recombinant lentiviruses were packaged and transfected into cancer cells in accordance with the manufacturer’s instructions. The cells were treated with 0.5 μg/mL puromycin to obtain stable clones. Plasmids were extracted using the TOOLS plasmid mini kit (BIOTOOLS, Taipei, Taiwan).

### Lipid peroxidation assay

The degree of lipid peroxidation was determined by measuring the MDA level by using a lipid peroxidation (MDA) assay kit (#ab118970, Abcam, Cambridge, UK). Whole-cell lysates were collected and reacted with thiobarbituric acid and incubated at 95° C for 60 min. They were then cooled in an ice bath for 10 min to generate an MDA–thiobarbituric acid adduct. The MDA–thiobarbituric acid adduct was quantified colorimetrically (OD = 532 nm).

### Immunohistochemistry staining

Clinical formalin-fixed, paraffin-embedded HCC tissue blocks were subjected to immunohistochemistry (IHC) staining. The tissues of patients with HCC were obtained from the Joint Biobank of Taipei Medical University. The tissues were cut into 4-μm sections, deparaffinized, and rehydrated before antigen retrieval by heating the tissue sections in citrate buffer (pH = 6.0). Endogenous peroxidase and alkaline phosphatase activity were blocked with 0.3% hydrogen peroxide in 95% ethanol for 5 min. The sections were incubated with the anti-Galectin-1 primary antibody at 4° C overnight. Subsequently, the sections were incubated with a secondary antibody conjugated to horseradish peroxidase polymer for 30 min at room temperature and washed three times for 10 min with phosphate-buffered saline. The sections were analyzed after diaminobenzidine was applied as a chromogen, and Mayer’s hematoxylin (Histolab) was used for counterstaining.

### Bioinformatics analysis

Details regarding Galectin-1, AXL, and MET expression in patients with HCC were obtained from the TCGA database by using the UALCAN data analysis portal (http://ualcan.path.uab.edu/) and subsequently analyzed. We conducted a Kaplan–Meier survival analysis for patients with HCC by using the PrognoScan database (http://dna00.bio.kyutech.ac.jp/PrognoScan/) based on the differential expression of Galectin-1, AXL, and MET.

### Statistical analysis

Student’s *t* test was used to identify between-group differences in continuous variables. All data are presented as means ± standard errors of the mean, and a *P* value of <0.05 indicated significant differences. All measurements were performed at least thrice and analyzed using GraphPad Prism software for variance analysis among three or more groups.

## Supplementary Material

Supplementary Figures
