# Supplementary material for: Galectin-1-mediated MET/AXL signaling enhances sorafenib resistance in hepatocellular carcinoma by escaping ferroptosis
Source: Aging (Albany NY). 2023 Jul 11;15(13):6503–25. doi: 10.18632/aging.204867 (PMC10373977; doi:10.18632/aging.204867)
Supplement: Supplementary Figures [file aging-15-204867-s001.pdf]

SUPPLEMENTARY FIGURES

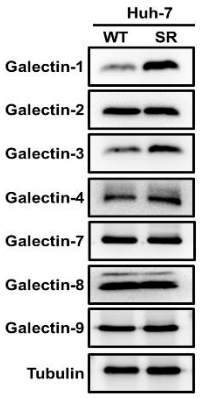

Supplementary Figure 1. **Examine the protein expression of the galectin family in Huh-7 and Huh-7/SR cells.** Protein expression of the galectin family (Galectin-1, -2, -3, -4, -7, -8, and -9) in Huh-7 and Huh-7/SR cells was analyzed by Western blotting.

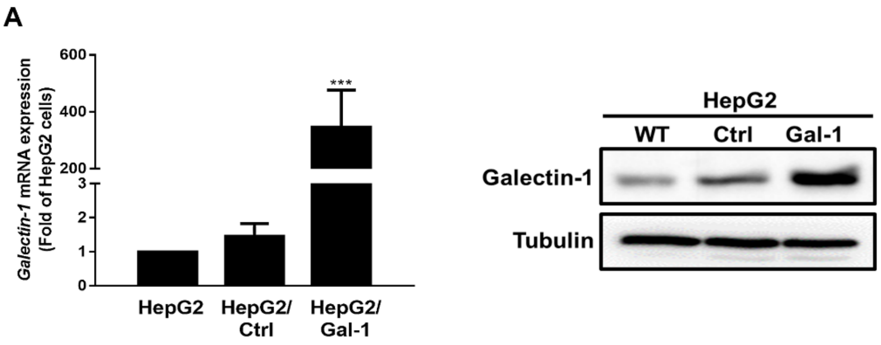

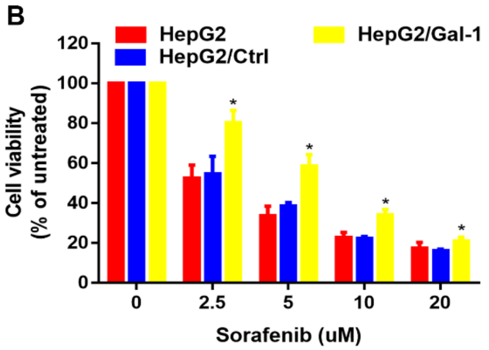

Supplementary Figure 2. Overexpression of Galectin-1 enhances sorafenib resistance in HCC cells. qRT-PCR and Western blotting analysis detected Galectin-1 (**A**) mRNA and (**B**) protein expression in HepG2 cells after Galectin-1 overexpression. (**B**) Cell viability of the indicated cells was measured using an MTT assay. Data are presented as means ± standard deviations. *$P < 0.05$, **$P < 0.01$, and ***$P < 0.001$ (Student's *t* test).

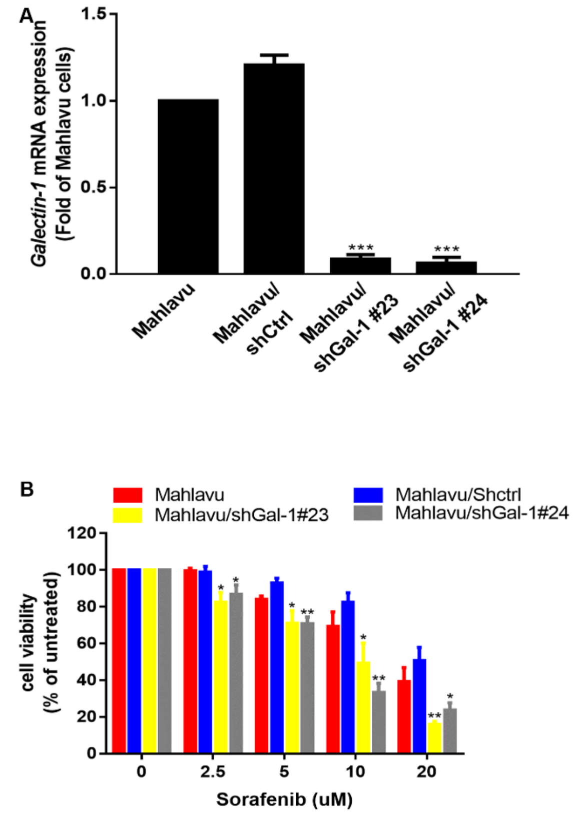

Supplementary Figure 3. Inhibition of Galectin-1 reduced sorafenib resistance in HCC cells. (**A**) qRT-PCR analysis detected Galectin-1 mRNA expression in Mahlavu cells after Galectin-1 knockdown. (**B**) Cell viability of the indicated cells was measured using an MTT assay. Data are presented as means ± standard deviations. *P < 0.05, **P < 0.01, and ***P < 0.001 (Student's *t* test).

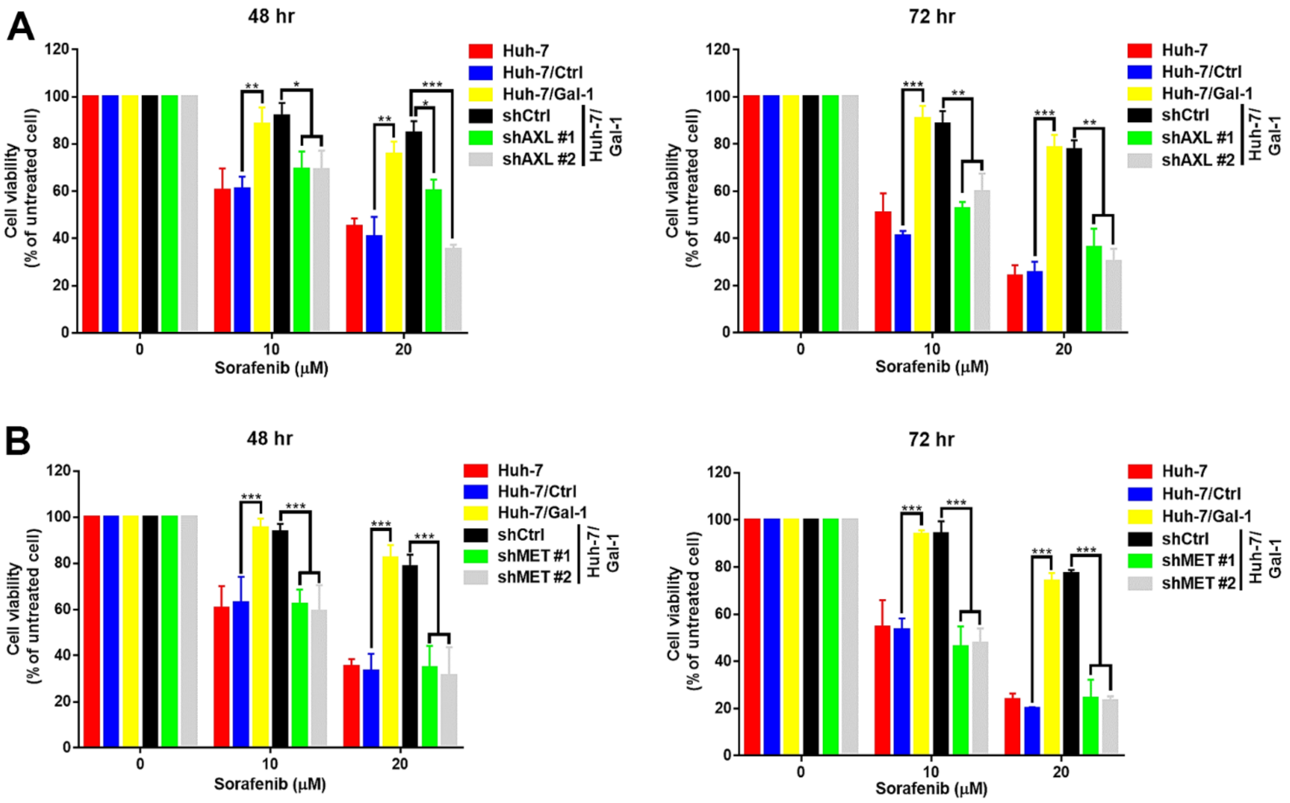

Supplementary Figure 4. **Knockdown of AXL and MET abolished Galectin-1 overexpression-induced sorafenib resistance.** Cell viability after knockdown of (**A**) AXL and (**B**) MET in Huh-7/Gal cells treated with sorafenib for 48 h was measured using the MTT assay. Data are presented as means ± standard deviations. *P < 0.05, **P < 0.01, and ***P < 0.001 (Student's t test).

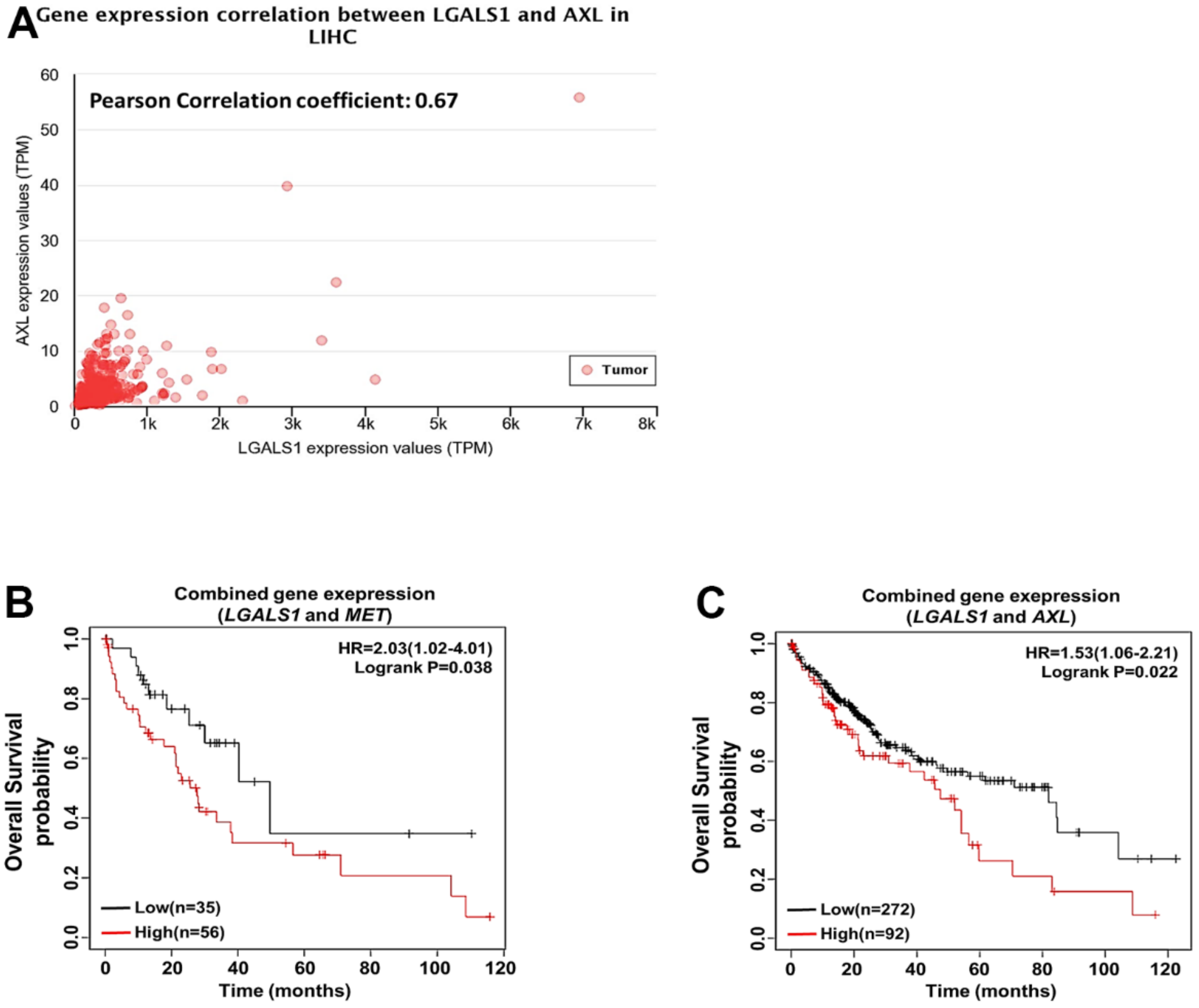

Supplementary Figure 5. MET and AXL reduced sorafenib resistance in HCC cells. (**A**) Galectin-1 was positively correlated with AXL in patients with HCC. (**B**) High Galectin-1 and MET expression were correlated with poor overall survival in patients with HCC. (**C**) High Galectin-1 and AXL expression was correlated with poor overall survival in patients with HCC.
